# Dialdehyde Starch Cross-Linked Collagen with Heparin Conjugation: Characterization and Feasibility Study for Osteochondral Tissue Repair

**DOI:** 10.3390/gels11110850

**Published:** 2025-10-24

**Authors:** Jason K. Lee, Jihye Baek, Shawn P. Grogan, Tae-Hoon Koo, Darryl D. D’Lima

**Affiliations:** 1 Shiley Center for Orthopaedic Research and Education at Scripps Clinic, 3550 John Hopkins Ct, Suite 110, San Diego, CA 92121, USA; jhbaek4a@gmail.com (J.B.); sgrogan@scripps.edu (S.P.G.); 2 D.med LLC, 111, Sagimakgol-ro, Jungwon-gu, Seongnam-si 13202, Gyeonggi-do, Republic of Korea; glenkoo@dmed.co.kr

**Keywords:** collagen, crosslinking, dialdehyde starch, heparin, growth factor, chondrocyte, hydrogel, scaffold, osteochondral, cartilage

## Abstract

Collagen is widely used in tissue engineering due to its excellent biocompatibility; however, its limited intrinsic mechanical strength restricts its application in load-bearing environments. This study introduces dialdehyde starch (DAS) as a biocompatible macromolecular cross-linker to enhance the mechanical integrity of collagen hydrogels. Collagen gels were cross-linked with DAS during neutralization under optimized conditions, resulting in a significant increase in compressive stiffness (up to ~125 kPa), thereby improving their suitability for mechanically demanding applications. Degradation studies of DAS-crosslinked collagen confirmed the long-term stability of the gel, while post-neutralization heparin incorporation improved bifunctionality, as evidenced by increased surface retention. FT-IR analysis confirmed the successful DAS cross-linking and heparin conjugation while preserving the native collagen structure. Bioactivity assays of DAS-crosslinked and heparin-conjugated collagen gel demonstrated enhanced chondrocyte migration in PDGF-BB-functionalized gels and improved cell viability, proliferation, and matrix deposition in TGF-β3-treated constructs. Preliminary ex vivo culture using a rabbit osteochondral defect model showed promising tissue integration and glycosaminoglycan accumulation. These results highlight the potential of DAS-crosslinked and heparin-conjugated collagen hydrogels as mechanically robust and biologically supportive scaffolds for osteochondral tissue engineering and regenerative medicine applications.

## 1. Introduction

Collagen is the most abundant structural protein in musculoskeletal tissues and organs, playing a vital role in maintaining their mechanical integrity and biological function [1,2,3]. Owing to its excellent biocompatibility, biodegradability, and low antigenicity, collagen has been widely utilized as a scaffold and carrier material in tissue engineering and regenerative medicine [4,5]. However, its intrinsic mechanical weakness remains a major limitation for broader applications, especially in load-bearing or structurally demanding environments [6,7]. To overcome this, various chemical modifications and cross-linking strategies have been developed to enhance the mechanical and structural properties of collagen [8,9,10].

Numerous cross-linking methods, including but not limited to glutaraldehyde, formaldehyde, EDC (1-ethyl-3-(3-dimethylaminopropyl) carbodiimide hydrochloride)-NHS (N-hydroxy succinimide), genipin, and UV-induced polymerization of methacrylate collagen, have been explored to improve collagen’s performance; however, achieving a balance between mechanical reinforcement and biological compatibility remains a challenge [11,12,13,14,15]. Traditional covalent cross-linking agents such as glutaraldehyde, formaldehyde, and carbodiimide-based systems, EDC-NHS, have been extensively employed, resulting in an enhanced physical stability of collagen. However, the cytotoxicity of residual formaldehyde and its potential to induce inflammatory responses pose significant limitations for in vivo applications [16]. Similarly, glutaraldehyde, despite its strong cross-linking ability, suffers from poor biocompatibility due to the presence of unreacted aldehyde groups [17,18]. EDC/NHS chemistry offers a less toxic alternative by facilitating the formation of amide bonds between carboxyl and amino groups in collagen, resulting in improved structural integrity and alignment of collagen fibrils. Nonetheless, these reactions often exhibit low cross-linking efficiency, require extended reaction times, and residual unreacted molecules may still interfere with cellular viability [19]. Alternatively, genipin, a naturally derived cross-linker, offers reduced cytotoxicity compared to glutaraldehyde but also suffers from limitations such as slow cross-linking kinetics and variability in cross-linked gel strength [20]. More recently, methacrylate-mediated photo-crosslinking has gained attention, especially in 3D bioprinting applications, where spatial control over gelation is critical [21,22,23,24]. This approach imparts significant mechanical strength to collagen hydrogels through UV-induced polymerization of methacrylate collagen [25,26,27]. However, residual acrylate groups may elicit inflammatory responses and compromise biocompatibility, making their clinical translation challenging [28,29,30]. In addition to chemical cross-linkers, various synthetic and natural polymers have been blended with collagen to enhance its functional properties [31,32,33,34,35]. While such combinations improve strength and elasticity, they introduce chemical, manufacturing, regulatory, and biological complexities that may hinder their clinical translations [7,36]. Therefore, developing a simplified, efficient, and cell-compatible method to enhance collagen’s mechanical properties remains a critical objective.

Despite these ongoing advances, it remains evident that none of the current collagen cross-linking strategies are yet sufficient to fully meet the stringent physical and mechanical requirements of load-bearing applications [4]. While improvements in stiffness, structural stability, and fibril alignment have been demonstrated, the levels of mechanical reinforcement achieved are often inadequate compared to native load-bearing tissues, such as bone, tendon, or articular cartilage [11,37,38]. Moreover, trade-offs in biocompatibility, processing time, and scalability further limit their translational potential [39]. Therefore, the development of a simplified, efficient, and truly cell-compatible cross-linking approach capable of delivering clinically relevant mechanical performance continues to be a critical unmet need in the field of collagen-based biomaterials.

Starch, particularly in its oxidized form, presents a promising alternative. Starch-based materials have long been used in bone graft fillers due to their plasticity and moldability [40,41,42,43]. Moreover, starch nanoparticles and nanocapsules have demonstrated excellent biocompatibility, mechanical performance, and even antimicrobial activity, making them attractive for scaffold applications in tissue engineering and drug delivery [44,45,46,47,48]. In this study, we investigated the use of periodate-oxidized starch, or dialdehyde starch (DAS), as a macromolecular cross-linker for collagen (DAS-COL) gels. DAS, with its high molecular weight and bifunctional aldehyde groups, forms intermolecular cross-links with collagen without disrupting its secondary structure. Unlike previous studies that primarily focused on freeze-dried or cryogel collagen scaffolds without cellular encapsulation [49], we aim to explore the effect of DAS on cell-laden collagen hydrogel. We hypothesize that tuning the concentration of DAS, solvent conditions, and neutralization buffers can not only enhance the physical integrity of the collagen network but also support an environment conducive to cell proliferation and new extracellular matrix deposition. We also hypothesize that the neutralization is a critical step in the crosslinking reaction, as it activates DAS-mediated crosslinking by adjusting the pH to physiological levels, enabling efficient interaction between DAS and collagen molecules.

In addition to DAS-COL gel, we examined the heparin-conjugated variant (DAS-COL-HEP), given our prior findings that heparin conjugation improves growth factor binding to collagen [50,51,52]. In addition to the gel form, we fabricated a dried, porous, cylindrical version of DAS-COL-HEP, and we applied it to a rabbit osteochondral ex vivo model to explore its potential for future in vivo animal models. Therefore, this study aims to characterize DAS-COL-HEP by examining the incorporation of heparin during DAS-COL preparation, assessing chemical structure changes after heparin conjugation and crosslinking, evaluating cell migration and proliferation in vitro via growth factor binding, and testing the clinical applicability of DAS-COL-HEP using a rabbit ex vivo model.

## 2. Results and Discussion

### 2.1. Compressive Stiffness Changes in DAS-COL by Conditional Neutralization Buffer

**Figure 1:**
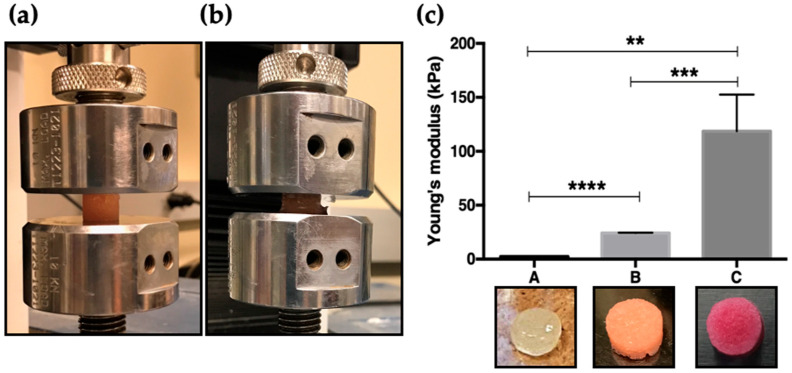
Compression test of a DAS-COL (5% *w*/*v* DAS and 4% *w*/*v* COL) gel block: (**a**) before compression; (**b**) after compression; (**c**) Young’s modulus of DAS-COL gel blocks (mean ± SD, *n* = 6) prepared with different concentrations of NaOH in the neutralization buffer: A (0.25 N), B (0.5 N), and C (0.75 N) NaOH conditions. Statistical significance was determined using one-way ANOVA with post hoc comparisons: ** *p* < 0.01, *** *p* < 0.001, **** *p* < 0.0001.

To investigate how the neutralization buffer composition influences the mechanical properties of DAS-COL hydrogels (Figure 1a,b), we formulated gels using different concentrations of NaOH (0.25 N, 0.5 N, and 0.75 N) in the neutralization buffer.

When the acidic collagen gel was mixed with DAS, reconstitution buffer, and conditional neutralization buffer sequentially, the color of the gel changed to beige-yellow, orange-red, and red-pink. Visual differences in gel opacity and coloration after the crosslinking step were observed by the phenol red in the reconstitution buffer, as shown in Figure 1c, with the gels becoming increasingly opaque and intensely colored as the NaOH concentration increased. The phenol red in the buffer indicates that the beige-yellow-colored gel is close to pH 6.0, the orange-red-colored gel is close to pH 7.0, and the red-pink-colored gel is close to pH 8.0. These changes are indicative of enhanced DAS cross-linking and stiffer gel formation by a specific alkaline pH range during the neutralization and reconstitution steps.

Mechanical testing revealed a strong positive correlation between NaOH concentration and hydrogel stiffness (Figure 1c). Gels reconstituted with 0.25 N NaOH exhibited minimal stiffness (~3 kPa, A), whereas those with 0.5 N and 0.75 N NaOH displayed significantly higher stiffness values (~30 kPa, B, and ~125 kPa, C, respectively). Statistical analysis confirmed that all pairwise comparisons were significant: A vs. B (**** *p* < 0.0001), B vs. C (*** *p* < 0.001), and A vs. C (** *p* < 0.01).

These findings demonstrate that increasing the concentration of NaOH in the neutralization buffer enhances cross-linking efficiency, thereby increasing the mechanical strength of DAS-COL hydrogels. Proper pH adjustment during reconstitution and neutralization steps is critical for tuning hydrogel physical properties and achieving the desired stiffness for specific applications.

### 2.2. Degradation Test of DAS-COL Hydrogels

**Figure 2:**
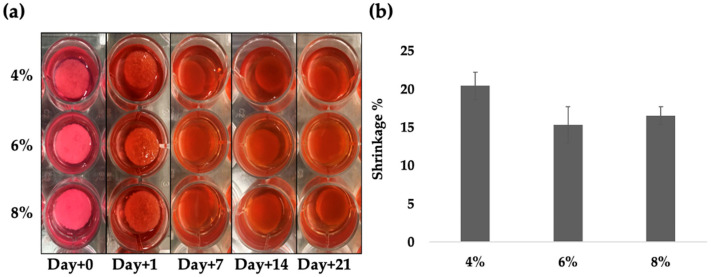
Degradation test of DAS-COL (5% *w*/*v* DAS and 4, 6, and 8% *w*/*v* COL) gel blocks: (**a**) Macro morphology at Days 0, 1, 7, 14, and 21; (**b**) Gel shrinkage analysis of different collagen concentration DAS-COL (mean ± SD, *n* = 3) from Day+0 to Day+21 in the basal media. Statistical significance was determined using one-way ANOVA.

The degradation of DAS-COL hydrogels with varying collagen concentrations (4%, 6%, and 8% *w*/*v*) was evaluated over 21 days in DMEM culture medium at 37 °C. Visual observations revealed shrinkage of the hydrogels, which stabilized by 2 weeks; however, no statistical differences were observed among the different collagen concentrations (Figure 2).

At Days 0 and 1 (average 0.85 ± 0.02 inches), all hydrogels maintained their structure and opacity. By Day 7 (average 0.70 ± 0.02 inches), DAS-COL gels exhibited slight degradation, showing gel block shrinkage. However, by Days 14 and 21, the gels maintained the size and shape with only minor changes in opacity and surface texture without further shrinkage. During the degradation test in the culture medium, the gel color changed to match that of the culture medium, suggesting that the pH of the DAS cross-linked collagen gel could be adjusted to a cell culture-compatible condition, while the gels maintained their structure in the neutralized pH environment without significant degradational changes.

### 2.3. Toluidine Blue Assay for Heparin Conjugated DAS-COL (DAS-COL-HEP)

**Figure 3:**
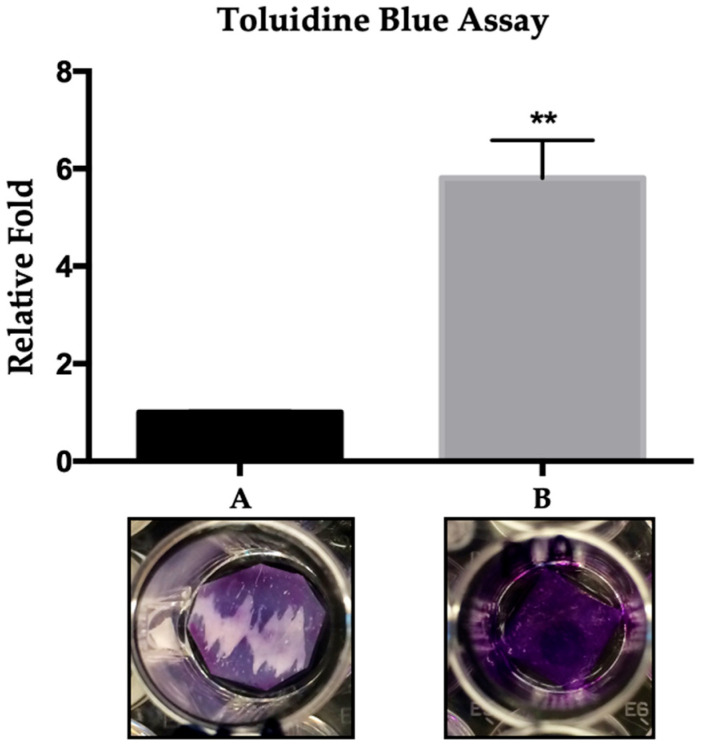
Toluidine blue assay for DAS-COL-HEP (5% *w*/*v* DAS and 4% COL) gels: Heparin sodium salt (0.1% *w*/*v*) was incorporated into DAS-COL (*n* = 3) either before neutralization (A) or after neutralization (B). Representative images of stained gels are shown below each corresponding bar. Data are presented as relative fold change (mean ± SD, *n* = 3). ** *p* < 0.001.

Heparin conjugation is commonly used to bind bioactive molecules (e.g., growth factors) to collagen. To assess how the timing of heparin incorporation affects its surface retention in DAS-COL hydrogels, we performed a toluidine blue binding assay. Hydrogels were prepared by adding heparin either before or after the neutralization step (in NaHCO_3_/NaOH/HEPES buffer) and compared as groups A (heparin mixed with post-crosslinked DAS-COL) and B (heparin mixed during DAS-COL preparation, followed by crosslinking), respectively (Figure 3).

After staining and extraction, the B gel (DAS-COL-HEP) group exhibited a significantly stronger absorbance signal than the A gel group, indicating increased heparin retention. Visually, the B gel appeared deeply and uniformly stained, whereas the A gel showed weak and patchy coloration. Quantitative analysis confirmed that the B gel retained approximately 6-fold more heparin than the A gel (*p* < 0.01). These results suggest that the method involving heparin mixing during DAS-COL preparation, followed by crosslinking, enhances the surface binding efficiency of heparin to collagen. Moreover, gels produced using this method showed increased porosity and stiffness in the gel structure (Figure S1).

### 2.4. FT-IR Analysis of DAS-Crosslinked and Heparin-Conjugated Collagen

**Figure 4:**
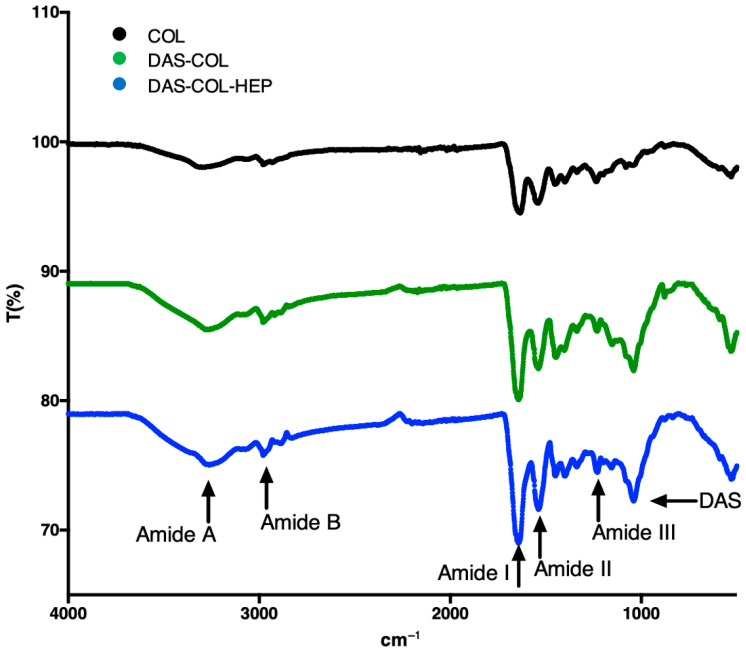
FT-IR analysis: FT-IR spectra of unmodified collagen (COL), dialdehyde starch cross-linked collagen (DAS-COL: 5% *w*/*v* DAS and 4% *w*/*v* COL), and heparin-functionalized DAS-COL (DAS-COL-HEP: 5% *w*/*v* DAS, 4% *w*/*v* COL, and 0.1% *w*/*v* HEP). Characteristic absorption bands corresponding to Amide A (~3300 cm^−1^), Amide B (~2920 cm^−1^), Amide I (~1650 cm^−1^), Amide II (~1550 cm^−1^), and Amide III (~1240 cm^−1^) are indicated (black arrows).

The FT-IR spectra of COL, DAS-COL, and DAS-COL-HEP samples reveal distinct changes, confirming the cross-linking reaction and heparin conjugation (Figure 4).

Characteristic amide bands are observed across the samples. The Amide A (~3300 cm^−1^) and Amide B (~2920 cm^−1^) bands, corresponding to N–H and C–H stretching vibrations, respectively, show slight shifts upon modification, indicating changes in the local chemical environment due to cross-linking [53].

The appearance and shifting of the Amide III band (associated with C–N stretching and N–H deformation) are particularly significant, as this band is sensitive to the degree of cross-linking [54]. The intensity of the Amide III band increases in the DAS-COL and DAS-COL-HEP samples, indicating enhanced cross-linking through the interaction of dialdehyde starch (DAS) with free amino groups in collagen (COL) [55]. This occurs via Schiff base formation, where NH_2_ groups are converted into C=N imine linkages, without disrupting the collagen’s native triple-helical structure [49].

The Amide II band (~1550 cm^−1^), originating from N–H bending coupled with C–N stretching, also shows shifts in the modified samples, further supporting successful chemical interaction [55]. Additionally, the emergence of peaks related to C–O stretching vibrations in DAS indicates the formation of hydrogen bonds in the cross-linked materials [49,55]. The observed spectral changes are consistent with effective cross-linking of collagen by DAS and subsequent heparin functionalization, comparable to modifications reported in prior studies [49,56,57].

Changes in intensity, rather than shifts in wavenumber, of characteristic collagen bands, particularly Amide III, support successful cross-linking via Schiff base formation between the aldehyde groups of DAS and the amino groups of COL, consistent with observations from the previous study [55]. Furthermore, the appearance and increased intensity of bands around ~1030 cm^−1^ in the DAS-COL and DAS-COL-HEP spectra indicate the presence of DAS-derived functional groups. Additional spectral features suggest hydrogen bonding interactions and successful incorporation of HEP into the biopolymer network [55].

### 2.5. Chondrocyte Migration Test of PDGF-BB Conjugated DAS-COL-HEP

**Figure 5:**
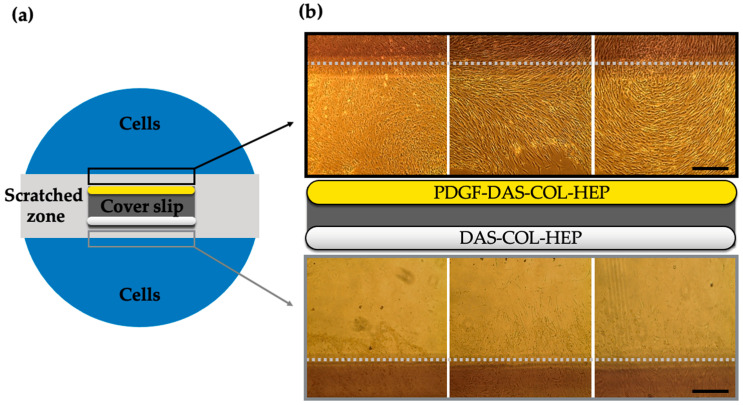
Scratch assay of growth factor conjugated DAS-COL-HEP (5% *w*/*v* DAS, 4% *w*/*v* COL, and 0.1% *w*/*v* HEP): (**a**) DAS-COL-HEP (thick white bar) and PDGF-BB (200 ng/mL) conjugated DAS-COL-HEP (thick yellow bar) were spread on the cover slip (dark gray rectangle). Bovine chondrocytes were cultured on a 10 cm culture dish (blue circle). The middle part of the cells was scratched with a cell lifter (3 cm wide). The gel spread cover slip was attached to the middle of the scratched zone (white rectangle) with a surgical dermal bond. (**b**) Microscopy of migrated cells after 3-day culture showed migrated cells near the PDGF binding DAS-COL-HEP (upper) and the DAS-COL-HEP (lower). The gray dotted line indicates the borderline of the scratched zone.

To evaluate the effect of chemotactic PDGF-BB (200 ng/mL) conjugated DAS-COL-HEP on cell migration, a scratch assay was designed (Figure 5a), and the migrated cells by DAS-COL-HEP with and without PDGF-BB conjugation were compared (Figure 5b).

The migrated cells were shown in the PDGF-DAS-COL-HEP group, while there were a few cells seen in the DAS-COL-HEP group. In the PDGF-DAS-COL-HEP condition (upper panel, yellow bar), a substantial number of chondrocytes had migrated into the scratched zone, visibly crossing the gray dotted line that marks the boundary of the original cell-free area. In contrast, the DAS-COL-HEP group (lower panel, white bar) exhibited significantly fewer migrated cells, with most cells remaining near the edge of the scratch zone and limited cellular infiltration into the central region. These observations indicate the efficacy of PDGF-BB binding to DAS-COL-HEP and that the presence of PDGF-BB in the hydrogel substantially promotes chondrocyte migration.

### 2.6. Chondrocyte Viability and Proliferation Test of TGF-β3 Conjugated DAS-COL-HEP

**Figure 6:**
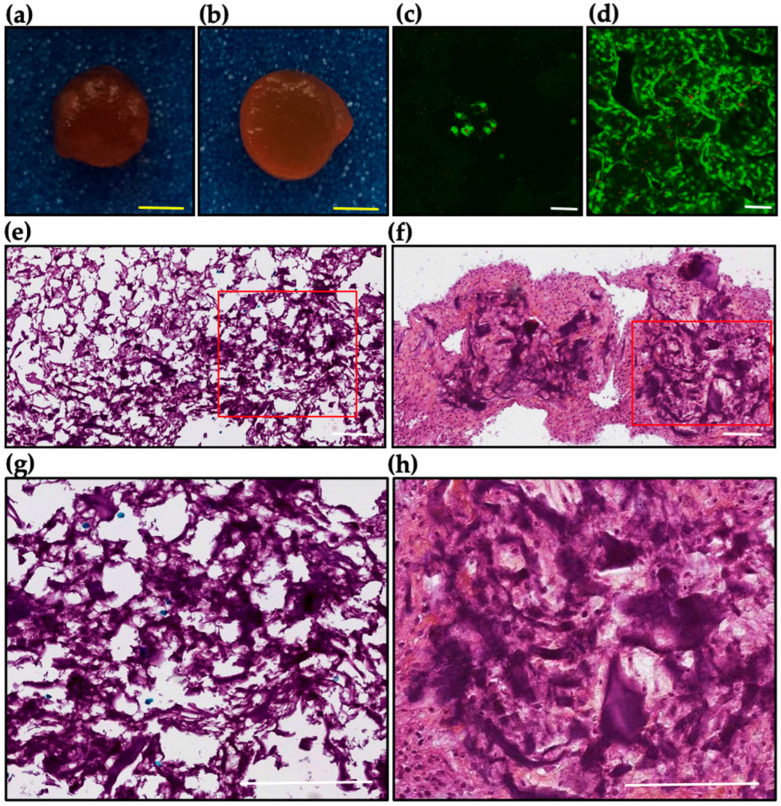
Macro- and micro morphologies of chondrocyte-encapsulated DAS-COL-HEP (5% *w*/*v* DAS, 4% *w*/*v* COL, and 0.1% *w*/*v* HEP) constructs with and without TGF-β3: Bovine chondrocytes were encapsulated in DAS-COL-HEP hydrogel and shaped into cylindrical gel blocks. These constructs were cultured for 3 weeks in either basal medium (control group) or medium supplemented with TGF-β3 (200 ng/mL; growth factor group): (**a**,**b**) Macroscopic morphology of constructs: (**a**) control, (**b**) growth factor group; (**c**,**d**) Cell morphology after fluorescent live/dead staining: (**c**) control, (**d**) growth factor group; (**e**,**f**) Microscopy of Safranin O staining: (**e**) control, (**f**) growth factor group; (**g**,**h**) Microscopy of Safranin O staining of red lined box at higher magnification: (**g**) control, (**h**) growth factor group. Yellow scale bars = 5 mm; white scale bars = 100 μm.

To investigate the effects of TGF-β3 on chondrocyte behavior and extracellular matrix formation, bovine chondrocytes were encapsulated in DAS-COL-HEP hydrogels and cultured for 3 weeks in either basal medium (control group) or medium supplemented with TGF-β3 (200 ng/mL; growth factor group). The cell culture environment with DAS-COL, DAS-COL-HEP, and TGF-β3-bound DAS-COL-HEP was characterized in the pilot experiment (Figure S2), and DAS-COL-HEP was selected as a control group of the in vitro culture experiment. The macro- and microscopic characteristics of the cell-gel constructs were assessed (Figure 6).

Macroscopic examination revealed notable differences between the two groups (Figure 6a,b). Constructs cultured with TGF-β3 (Figure 6b) appeared denser and more compact than the control constructs (Figure 6a), suggesting enhanced tissue remodeling and matrix deposition under growth factor stimulation.

Fluorescent live/dead staining demonstrated live cells in both groups. However, a higher cell density was observed in the growth factor-treated scaffolds (Figure 6d) in comparison to the control group after 3 weeks (Figure 6c). The cell density in the gel was gradually decreased in the DAS-COL-HEP gel group, while the cell density in the TGF-β3-supplemented DAS-COL-HEP was slightly increased (Figure S3).

Microscopic histology image using Safranin O staining (Figure 6e,f) further supported the beneficial effect of TGF-β3. Constructs in the growth factor group (Figure 6f,h) showed intense staining, reflecting substantial glycosaminoglycan (GAG) accumulation, a hallmark of cartilage matrix formation. The control group (Figure 6e,g) exhibited weaker and more diffuse staining, indicating limited GAG production in the absence of TGF-β3. Collectively, these results demonstrate that TGF-β3 treatment significantly enhances chondrocyte viability, maintaining chondrogenic morphology and new extracellular matrix deposition within the DAS-COL-HEP hydrogel constructs.

### 2.7. Ex Vivo Evaluation of DAS-COL-HEP Blocks in a Rabbit Osteochondral Knee Model

**Figure 7:**
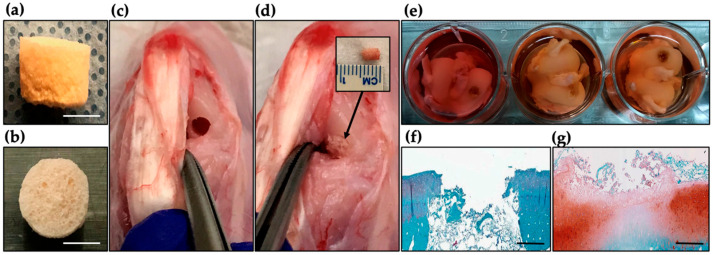
Ex vivo culture of lyophilized DAS-COL-HEP (5% *w*/*v* DAS, 4% *w*/*v* COL, and 0.1% *w*/*v* HEP) block in a rabbit osteochondral defect: (**a**) Side view; (**b**) top view of the lyophilized DAS-COL-HEP gel block; (**c**) Surgically created cylindrical defect in rabbit femoral articular cartilage before collecting the explant; (**d**) Insertion of the DAS-COL-HEP block into the defect site (5 mm diameter and 5 mm depth); (**e**) Retrieved femoral condyle explants (*n* = 3) were cultured for 3 weeks in the basal media; (**f**) Safranin-O staining of an untreated (empty) defect; (**g**) Safranin-O staining of a defect treated with the DAS-COL-HEP block after 3-week culture (White bars = 5 mm; Black bars = 1 mm).

Ex vivo culture was conducted to assess the potential of the lyophilized DAS-COL-HEP block for osteochondral tissue repair. Cylindrical osteochondral defects were created in rabbit femoral condyles and implanted with lyophilized DAS-COL-HEP. The femoral condyles were harvested and cultured ex vivo in basal media for 3 weeks. (Figure 7a–d). Explants were retrieved after 3-week culture in basal medium to assess tissue response and matrix deposition (Figure 7e).

Histological analysis using Safranin O staining revealed clear differences between untreated and treated defects. The untreated (empty) defect (Figure 7f) showed minimal staining, indicating poor cartilage matrix formation and limited GAG content. In contrast, the defect treated with the DAS-COL-HEP block (Figure 7g) exhibited moderate to strong staining within the defect region, suggesting gel block integration with a host tissue, enhanced GAG deposition, and early cartilage-like tissue formation. The stained region appeared more organized and partially integrated with the surrounding native cartilage tissue. These preliminary findings support the biocompatibility and cartilage-regenerative potential of DAS-COL-HEP blocks, indicating their promise as a scaffold for osteochondral tissue repair in vivo.

### 2.8. Discussion

This study presents a novel approach to improving the mechanical and biological performance of collagen hydrogels through cross-linking with dialdehyde starch (DAS) and subsequent heparin functionalization for better growth factor binding. The primary goal was to develop a biocompatible, structurally robust scaffold suitable for load-bearing and regenerative medicine applications, particularly in osteochondral tissue repair. DAS-COL and DAS-COL-HEP hydrogels were thoroughly characterized via mechanical and bioactivity assays. Mechanical integrity, degradation, and collagen structure were assessed through compressive stiffness measurements and FT-IR spectroscopy. For DAS-COL-HEP, additional analyses included heparin retention, growth factor binding, and cellular responses using toluidine blue quantification, in vitro migration and viability assays, and a rabbit osteochondral defect model. These results confirm that both hydrogel platforms support robust physical properties and relevant biological functions for cartilage tissue engineering applications. Our results validate the working hypothesis that DAS, as a macromolecular cross-linker, can significantly enhance the mechanical stiffness and structural stability of collagen hydrogels (Video S1) while maintaining a cell-permissive environment. Additionally, the integration of heparin and growth factors (PDGF-BB, TGF-β3) imparts bio-functionality that supports host cell migration or cell proliferation in the DAS-COL structures as porous sponge or gel types, thus extending the scope of these hydrogels to cartilage tissue engineering. One of the most important considerations in developing advanced biomaterials for tissue repair and regeneration is ensuring biocompatibility, which is essential for mitigating regulatory challenges and enhancing the feasibility of clinical applications in a timely manner.

Previous studies have explored various cross-linking strategies to enhance the mechanical strength of collagen, including glutaraldehyde, EDC/NHS, and Genipin. While effective in reinforcing the collagen matrix, these methods often suffer from limitations such as cytotoxicity, low efficiency, or poor scalability. DAS, in contrast, offers a biocompatible and efficient cross-linking mechanism via Schiff base formation between aldehyde and amine groups, enabling the formation of a mechanically reinforced matrix without compromising cellular compatibility. Our compression testing demonstrated a significant increase in Young’s modulus (~125 kPa with 0.75 N NaOH), which is a substantial improvement over unmodified collagen gel and comparable or superior to other cross-linkers reported in the literature [58,59,60]. This reinforces DAS’s potential as a clinically translatable alternative, especially for applications requiring enhanced mechanical performance (Videos S2 and S3).

The effect of DAS cross-linking on hydrogel stability was also evident in the 21-day stability study. Despite minor shrinkage in the early culture, the overall construct integrity was maintained across all collagen concentrations tested. This stability is critical for in vivo applications, particularly for cartilage repair, where prolonged residence time is necessary to support cellular remodeling and tissue integration. Importantly, FT-IR analysis confirmed successful cross-linking and preservation of the collagen’s triple helical structure, supporting the material’s structural compatibility with native extracellular matrix (ECM) components.

Incorporation of heparin into the hydrogel matrix has the potential to enhance the bioactivity of the DAS-COL gel. Our findings demonstrated that post-neutralization addition of heparin significantly improved its surface retention, as evidenced by toluidine blue staining and quantification. This is likely due to the enhanced electrostatic and chemical interactions between heparin and the positively charged collagen/DAS matrix under post-neutralization conditions. Heparin’s role in stabilizing and presenting growth factors is well-established [50], and its integration here serves to potentiate the scaffold’s therapeutic functionality without altering the cross-linking structure. Besides the enhancement of growth factor binding affinity, cross-linked heparin to collagen-based biomaterials increased the mechanical strength [61]. Therefore, heparin not only affected controlling the release of growth factor from the collagen gel but also improved the mechanical properties of the gel due to its cross-linking with collagen components [62].

Based on the chemical structure of unmodified collagen, DAS cross-linking increased the intensity of the amide I and II bands; however, no shifts in wavenumber were observed. This indicates that DAS cross-linking formed a new covalent imine bond (C=N) from the Schiff base reaction, and this appeared in the range of 1640–1690 cm^−1^, the amide I band. The amide II band of collagen, centered around 1540 cm^−1^, arises from the N-H bending and C-N stretching vibrations. The changes in its intensity reflect conformational changes in the protein backbone due to the chemical cross-links. Importantly, research showed that cross-linking with DAS does not disrupt the native triple helix conformation of collagen [55,57]. The enhanced physical properties of collagen by DAS cross-linking were obtained without altering its chemical structure. Additionally, the heparin-conjugated DAS-COL (DAS-COL-HEP) did not show any changes in the FT-IR spectra, suggesting that heparin conjugation does not affect the chemical structure of collagen. Therefore, the FT-IR data suggest that DAS cross-linking reinforces collagen structurally by increasing the chemical connectivity, while the heparin conjugation introduces a growth factor binding functionality without disrupting the intrinsic structure of the collagen molecules [63,64].

Biological assays further confirmed the multifunctionality of DAS-COL-HEP scaffolds. The PDGF-BB-conjugated hydrogels significantly enhanced chondrocyte migration in a scratch assay, which is a critical prerequisite for defect repopulation and tissue integration. Similarly, encapsulated chondrocytes cultured with TGF-β3 displayed improved proliferation and robust glycosaminoglycan (GAG) deposition—hallmarks of chondrocyte proliferation, condensation, and maintenance [65,66,67]. These results align with previous reports on the role of PDGF-BB in cell recruitment and TGF-β3 in chondrocyte proliferation [50,52,65,68], indicating that DAS-COL-HEP is a conductive platform for both paracrine and autocrine cellular responses.

The preliminary ex vivo rabbit model further demonstrated the translational promise of this scaffold. Compared to untreated defects, DAS-COL-HEP-implanted sites showed improved tissue integration, suggesting early matrix integration and cartilage-like tissue regeneration by the scaffold. Enhancing the early integration and mechanical stability of osteochondral scaffolds is crucial for successful defect repair and preventing future osteoarthritis [69]. Achieving strong integration early after implantation is essential for effective load transfer and long-term stability. Although further in vivo studies with larger sample sizes and longer follow-up are needed, these early findings support the scaffold’s integrative healing potential.

Future research directions should aim to optimize the degradation rate and growth factor delivery profiles of the DAS-COL-HEP scaffolds. In the practical applications, the sterilization of collagen-based scaffolds can present significant challenges, as certain methods may compromise their mechanical properties, biochemical integrity, or biocompatibility; therefore, stability of post-sterilized DAS-COL-HEP scaffolds and controlled degradation aligned with the rate of neo tissue formation is essential for avoiding premature loss of mechanical support. Cell proliferation was enhanced within the growth factor-enriched gel environment. Accordingly, further investigation is warranted to establish methodologies for isolating endogenous growth factors from patient-derived blood or bone marrow and for achieving their efficient and reproducible immobilization onto DAS-COL-HEP implants.

Moreover, engineering dual or sequential delivery systems for multiple growth factors (e.g., combining TGF-β3 and IGF-1) could further enhance regenerative outcomes. Investigating the immune response in vivo and validating the scaffold in larger animal models are necessary steps before clinical translation. Additionally, robust long-term mechanical stability testing, such as fatigue or cyclic loading tests, is needed. And lastly, adapting the DAS-COL-HEP system for 3D bioprinting may open new possibilities for personalized osteochondral graft fabrication.

## 3. Conclusions

This study demonstrates that DAS-crosslinked, heparin-functionalized collagen hydrogels represent a promising class of bioengineered scaffolds with improved mechanical strength, growth factor binding capacity, and support for cell proliferation. By combining structural robustness with growth factor delivery and cellular compatibility, these scaffolds address key limitations of traditional collagen hydrogels and offer new avenues for load-bearing tissue engineering applications, particularly in articular cartilage or osteochondral defect repair and regeneration.

## 4. Materials and Methods

### 4.1. Preparation of Dialdehyde Starch Crosslinked Collagen (DAS-COL)

**Figure 8:**
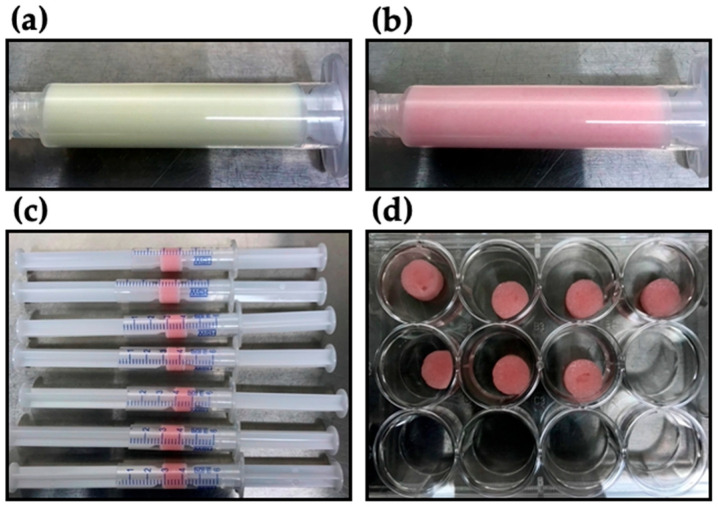
Dialdehyde starch (5% *w*/*v*) mixed collagen gel (4% *w*/*v*): (**a**) before neutralization; (**b**) after neutralization with 0.75 N NaOH buffer; (**c**) the extruded gel of 1 mL was loaded on the syringe barrel to form a cylindrical shape; (**d**) the gel block was placed in a 12-well plate for testing.

Collagen (D-med, Seongnam-si, Republic of Korea) extracted from porcine skin was dissolved with 0.1 M acetic acid to make 4% *w*/*v* concentration. Dialdehyde starch (Monomer Polymer & Dajac Labs, Ambler, PA, USA) of 5% (*w*/*v*), as a cross-linker, was dissolved in distilled water. Neutralization buffer was made of HEPES (200 mM) and NaHCO_3_ (2.2% *w*/*v*) with 0.75N NaOH. 10 × DPBS, including phenol red (0.5% *v*/*v*), was used for the reconstitution buffer. Phenol red was used as a pH indicator. All the chemical reagents for the buffer were purchased from Sigma Aldrich (St. Louis, MO, USA). For making a DAS-COL gel block, collagen gel was mixed with 10% volume of DAS solution. Each of the DAS-COL mixture, reconstitution buffer, and neutralization buffer was sequentially mixed in an 8:1:1 volume ratio. The reconstituted and neutralized DAS-COL was promptly transferred to the syringe barrel for the cross-linking reaction and to make a cylindrical shape (Figure 8). The formed gel blocks were placed in a multiplate well before use.

### 4.2. Compression Test of DAS-COL Gel Blocks

The compressive behavior of DAS-COL with various neutralization conditions was evaluated using a mechanical testing system. DAS-COL (5% *w*/*v* DAS and 4% *w*/*v* collagen) was prepared and neutralized under different conditions of NaOH concentrations, such as 0.25 N, 0.5 N, and 0.75 N, to see how the neutralization conditions affect the DAS cross-linking efficacy. Different conditional DAS-COL gel blocks (*n* = 6) were prepared for the compression test. The compression test was conducted using a custom-designed device consisting of a linear actuator (SMAC, Carlsbad, CA, USA), a 50 g load cell (FUTEK, Irvine, CA, USA) with a steel plunger having a flat surface for compression, and LabVIEW 2015 software (National Instruments, Austin, TX, USA) for movement control and data acquisition on a laptop. Each gel block was placed on the 80 mm-diameter plastic dish for the compression test. The height was measured with a vernier caliper. Preload was ensured in the negative Z direction when the gel block reached the preload value, which was 0.005 N, and the movement stopped. A 5% of original height step compression was applied to the gel subsequently, and the compression load data were recorded to calculate Young’s modulus.

### 4.3. Degradation Test of DAS-COL Gel Blocks

The shrinkage and dissolution behaviors of various concentrations of DAS-COL were assessed using culture media. DAS-COL gel blocks were made from different concentrations of DAS-COL (4%, 6%, and 8% *w*/*v*), neutralized with 0.75 N NaOH reconstitution buffer to investigate degradation changes in DAS-COL by different collagen concentrations. The gel blocks were incubated in DMEM containing 10% calf serum and 1% penicillin-streptomycin-glutamine (basal media) at 37 °C. Macro morphology and size changes in the gel block were compared at Days 0, 1, 7, 14, and 21. The given formula determined the percent shrinkage:

Shrinkage %=(L0−Lt)L0 × 100

where L0 is the initial diameter of the hydrogel and Lt is the diameter of the gel at time t (days).

### 4.4. Toluidine Blue Assay of Heparin-Conjugated DAS-COL (DAS-COL-HEP)

Quantitative characterization of heparin-conjugated DAS-COL (DAS-COL-HEP) was evaluated with the Toluidine blue assay. To conjugate heparin into the DAS-COL gel (5% *w*/*v* DAS and 4% *w*/*v* collagen), heparin sodium salt (0.1% *w*/*v*) was mixed with DAS-COL either before or after neutralization with 0.75 N NaOH buffer. Each conditional gel (*n* = 3) was plated on the hydrophobic PLA sheet (1 cm × 1 cm) and incubated for 4 h. The sheets were then stained with 0.4 mg/mL toluidine blue to detect the presence of heparin. After triple washing with distilled water, the bound dye was extracted using a solution of 0.1 M NaOH (20% *v*/*v*) and absolute ethanol (80% *v*/*v*). Absorbance of the extracted dye was measured using a microplate reader to quantify relative heparin content.

### 4.5. FT-IR of COL, DAS-COL, and DAS-COL-HEP

Chemical characterization of DAS-COL and DAS-COL-HEP was assessed with a Fourier Transform Infrared (FT-IR) analysis. To investigate possible changes in the chemical structure of collagen, resulting in its interaction with dialdehyde starch and heparin, unmodified collagen (COL), dialdehyde starch-crosslinked collagen (DAS-COL), and heparin-functionalized DAS-COL (DAS-COL-HEP) were analyzed using an FT-IR spectrometer (PerkinElmer FTIR, PerkinElmer, Waltham, MA, USA) equipped with an attenuated total reflectance accessory. Samples were lyophilized and ground into fine powders before measurement. Spectra were collected in the range of 4000–500 cm^−1^ with a resolution of 4 cm^−1^, and 32 scans were averaged per sample to improve the signal-to-noise ratio. Background spectra were recorded and automatically subtracted. All spectra were processed using the instrument’s proprietary software. Characteristic absorption bands corresponding to collagen’s protein backbone were identified, including Amide A (~3300 cm^−1^), Amide B (~2920 cm^−1^), Amide I (~1650 cm^−1^), Amide II (~1550 cm^−1^), and Amide III (~1240 cm^−1^) [54]. Shifts or changes in peak intensity were used to confirm cross-linking by dialdehyde starch and subsequent functionalization with heparin [55,70].

### 4.6. Cell Scratch Assay of PDBF-BB-Conjugated DAS-COL-HEP

The cell migration effect of angiogenic growth factor-conjugated DAS-COL-HEP was evaluated with the custom-designed cell scratch assay. Confluent bovine chondrocytes (5 × 10^6^ cells, Passage #2, Cell Applications, Inc., San Diego, CA, USA) were maintained on a 10 cm culture dish for the scratch assay. A linear scratch was introduced across the central region of the cell monolayer using a cell lifter (3 cm wide). Each of DAS-COL-HEP and DAS-COL-HEP, including PDGF-BB (200 ng/mL, rhPDGF-BB, R&D Systems, Minneapolis, MN, USA), was spread on the cover slip. The growth factor concentration was applied under the same conditions as our previous study [50]. The cover slip was attached to the middle of the scratched zone with a surgical dermal bond. Migrated cells were visualized through the microscope after a 3-day culture.

### 4.7. Cell Encapsulated DAS-COL-HEP in the TGF- β3 Growth Media

The cell viability and proliferation effects of growth factor-conjugated DAS-COL-HEP were evaluated with a live-dead cell assay and histology assessment. The chondrocytes (2 × 10^6^ cells/mL) were also encapsulated in DAS-COL-HEP gel, and the gel of 1 mL was shaped into a block. The chondrocytes encapsulated DAS-COL-HEP gel blocks were cultured for 3 weeks in either basal medium or medium supplemented with TGF-β3 (200 ng/mL, rhTGF-β3, PeproTech Inc., Cranbury, NJ, USA). The growth factor concentration was applied under the same conditions as our previous study [52]. After the culture, the gross morphology of the gel constructs was compared, and viable cells within the constructs were detected using Calcein-AM and Ethidium Homodimer-1 (Live/Dead kit, Life Technologies, Carlsbad, CA, USA) according to the manufacturer’s protocol. The staining was intended to qualitatively evaluate cell distribution, density, and general viability with treatment conditions. The cultured gel blocks were fixed in Z-fix (Anatech, San Jose, CA, USA) for 4 h. All samples were embedded in paraffin. Sections (5–7 μm thick) were stained for morphological analysis with Safranin O Fast Green to assess cellular and glycosaminoglycan distribution.

### 4.8. Ex Vivo Culture of DAS-COL-HEP Gel Block Implanted Rabbit Osteochondral Knee

A feasibility test for osteochondral defect healing with DAS-COL-HEP was designed with a rabbit ex vivo model. All animal experiments were performed in compliance with protocols approved by the Institutional Animal Care and Use Committee at The Scripps Research Institute. For the preliminary study of the rabbit implantation model, rabbits were sacrificed to collect the osteochondral knee after the implantation. Lyophilized DAS-COL-HEP blocks (n = 3) were implanted into a rabbit medial femoral condyle after making an osteochondral defect (5 mm diameter and 5 mm depth) with a biopsy punch. The knee blocks were separated from the distal femoral and proximal tibial bone cuts with a reciprocating bone saw (Stryker, Portage, MI, USA). The collected DAS-COL-HEP block implanted knee blocks were cultured with basal media for 3 weeks. After the culture, the knee blocks were fixed in Z-fix for 3 days, and decalcified in TBD-2 (Shandon, Pittsburgh, PA, USA) until being cut with a surgical blade. All samples were embedded in paraffin. Sections (5–7 μm thick) were stained with Safranin O Fast Green to assess tissue integration. The microscopic histology images were compared with an empty defect group and a DAS-COL-HEP block implantation group.

### 4.9. Statistical Analysis

Data represent mean and standard error of mean, from at least 3 to 4 replicate experiments, each performed in triplicate. The statistical significance of differences in compression test, gel degradation test, and cell viability test was determined using One-way ANOVA, with post hoc comparisons. Toluidine blue quantification was analyzed by an unpaired *t*-test. Results with * = *p* < 0.05 (95% CI, confidence interval), ** = *p* < 0.01 (99% CI), *** = *p* < 0.001 (99.9% CI), **** = *p* < 0.0001 (99.99% CI) were considered statistically significant.

## 5. Patents

US20230381377A1 has been published, resulting from the work reported in this manuscript.

## Data Availability

The data that support the findings of this study are available from the corresponding author upon reasonable request.

## Supplementary Materials

The following supporting information can be downloaded at: https://www.mdpi.com/article/10.3390/gels11110850/s1, Figure S1: DAS-COL-HEP with different porosity and stiffness under various heparin conjugation conditions; Figure S2: Pilot experiment of co-cultured bovine chondrocytes (1 × 10^7^ cells/mL) with different DAS-COL gel environments over 3 days; Figure S3: Co-cultured bovine chondrocytes (1 × 10^7^ cells/mL) in DAS-COL-HEP gels with or without TGF-β3 (200 ng/mL); Video S1: Cutting a gel block; Video S2: Pressing a sponge; Video S3: Injected gel into an articular cartilage defect.

## Abbreviations

The following abbreviations are used in this manuscript:

DAS	Dialdehyde Starch
COL	Collagen
HEP	Heparin
FT-IR	Fourier Transform Infrared (FT-IR) spectrometry
EDC	1-ethyl-3-(3-dimethylaminopropyl) carbodiimide hydrochloride
NHS	N-hydroxy succinimide
PDGF	Platelet-Derived Growth Factor
TGF	Transforming Growth Factor
DMEM	Dulbecco’s Modified Eagle’s Medium
GAG	Glycosaminoglycan
TBD	Decalcifier

## References

[1] Zhu J., Li Z., Zou Y., Lu G., Ronca A., D’Amora U., Liang J., Fan Y., Zhang X., Sun Y. (2022). Advanced application of collagen-based biomaterials in tissue repair and restoration. J. Leather Sci. Eng..

[2] Mathew-Steiner S.S., Roy S., Sen C.K. (2021). Collagen in Wound Healing. Bioengineering.

[3] Wang H. (2023). The Potential of Collagen Treatment for Comorbid Diseases. Polymers.

[4] Dong C., Lv Y. (2016). Application of Collagen Scaffold in Tissue Engineering: Recent Advances and New Perspectives. Polymers.

[5] Gajbhiye S., Wairkar S. (2022). Collagen fabricated delivery systems for wound healing: A new roadmap. Biomater. Adv..

[6] Zhang D., Wu X., Chen J., Lin K. (2018). The development of collagen based composite scaffolds for bone regeneration. Bioact. Mater..

[7] Zheng L., Tseomashko N., Voronova A., Vasil’kov A., Hu X., Wang X. (2024). Recent advances of collagen composite biomaterials for biomedical engineering: Antibacterial functionalization and 3D-printed architecturalization. Collagen Leather.

[8] Sarrigiannidis S.O., Rey J.M., Dobre O., González-García C., Dalby M.J., Salmeron-Sanchez M. (2021). A tough act to follow: Collagen hydrogel modifications to improve mechanical and growth factor loading capabilities. Mater. Today Bio.

[9] Li T., Zhou Z., Xie Y., Cai W., Zhu X., Jia Y., Zhang Z., Xu F., Huang G. (2025). Engineering strong and tough collagen hydrogels and tissue constructs via twisting and crosslinking. Cell Rep. Phys. Sci..

[10] Ye B., Wu B., Su Y., Sun T., Guo X. (2022). Recent Advances in the Application of Natural and Synthetic Polymer-Based Scaffolds in Musculoskeletal Regeneration. Polymers.

[11] Jiang Y.H., Lou Y.Y., Li T.H., Liu B.Z., Chen K., Zhang D., Li T. (2022). Cross-linking methods of type I collagen-based scaffolds for cartilage tissue engineering. Am. J. Transl. Res..

[12] Islam M.M., AbuSamra D.B., Chivu A., Argüeso P., Dohlman C.H., Patra H.K., Chodosh J., González-Andrades M. (2021). Optimization of Collagen Chemical Crosslinking to Restore Biocompatibility of Tissue-Engineered Scaffolds. Pharmaceutics.

[13] Angele P., Abke J., Kujat R., Faltermeier H., Schumann D., Nerlich M., Kinner B., Englert C., Ruszczak Z., Mehrl R. (2004). Influence of different collagen species on physico-chemical properties of crosslinked collagen matrices. Biomaterials.

[14] Powell H.M., Boyce S.T. (2006). EDC cross-linking improves skin substitute strength and stability. Biomaterials.

[15] Adamiak K., Sionkowska A. (2020). Current methods of collagen cross-linking: Review. Int. J. Biol. Macromol..

[16] Parhi R. (2017). Cross-Linked Hydrogel for Pharmaceutical Applications: A Review. Adv. Pharm. Bull..

[17] Gough J.E., Scotchford C.A., Downes S. (2002). Cytotoxicity of glutaraldehyde crosslinked collagen/poly(vinyl alcohol) films is by the mechanism of apoptosis. J. Biomed. Mater. Res..

[18] St Clair M.B., Bermudez E., Gross E.A., Butterworth B.E., Recio L. (1991). Evaluation of the genotoxic potential of glutaraldehyde. Environ. Mol. Mutagen..

[19] Shepherd D.V., Shepherd J.H., Ghose S., Kew S.J., Cameron R.E., Best S.M. (2015). The process of EDC-NHS Cross-linking of reconstituted collagen fibres increases collagen fibrillar order and alignment. APL Mater..

[20] Kawamura T., Yunoki S., Ohyabu Y., Uraoka T., Muramatsu K. (2021). Crosslinking Efficacy and Cytotoxicity of Genipin and Its Activated Form Prepared by Warming It in a Phosphate Buffer: A Comparative Study. Materials.

[21] Wang H., Wan J., Zhang Z., Hou R. (2024). Recent advances on 3D-bioprinted gelatin methacrylate hydrogels for tissue engineering in wound healing: A review of current applications and future prospects. Int. Wound J..

[22] Zennifer A., Manivannan S., Sethuraman S., Kumbar S.G., Sundaramurthi D. (2022). 3D bioprinting and photocrosslinking: Emerging strategies & future perspectives. Biomater. Adv..

[23] Tan G., Xu J., Yu Q., Zhang J., Hu X., Sun C., Zhang H. (2022). Photo-Crosslinkable Hydrogels for 3D Bioprinting in the Repair of Osteochondral Defects: A Review of Present Applications and Future Perspectives. Micromachines.

[24] Klotz B.J., Gawlitta D., Rosenberg A., Malda J., Melchels F.P.W. (2016). Gelatin-Methacryloyl Hydrogels: Towards Biofabrication-Based Tissue Repair. Trends Biotechnol..

[25] Ali S.M., Patrawalla N.Y., Kajave N.S., Brown A.B., Kishore V. (2022). Species-Based Differences in Mechanical Properties, Cytocompatibility, and Printability of Methacrylated Collagen Hydrogels. Biomacromolecules.

[26] Duymaz D., Karaoğlu İ.C., Kizilel S. (2025). Effect of Photoinitiation Process on Photo-Crosslinking of Gelatin Methacryloyl Hydrogel Networks. bioRxiv.

[27] Brinkman W.T., Nagapudi K., Thomas B.S., Chaikof E.L. (2003). Photo-cross-linking of type I collagen gels in the presence of smooth muscle cells: Mechanical properties, cell viability, and function. Biomacromolecules.

[28] Alizadehgharib S., Östberg A.K., Dahlgren U. (2017). Effects of the methacrylate/acrylate monomers HEMA, TEGDMA, DEGDA, and EMA on the immune system. Clin. Exp. Dent. Res..

[29] Lugović-Mihić L., Filija E., Varga V., Premuž L., Parać E., Tomašević R., Barac E., Špiljak B. (2024). Unwanted Skin Reactions to Acrylates: An Update. Cosmetics.

[30] Xia D., Chen J., Zhang Z., Dong M. (2022). Emerging polymeric biomaterials and manufacturing techniques in regenerative medicine. Aggregate.

[31] Shiroud Heidari B., Ruan R., Vahabli E., Chen P., De-Juan-Pardo E.M., Zheng M., Doyle B. (2023). Natural, synthetic and commercially-available biopolymers used to regenerate tendons and ligaments. Bioact. Mater..

[32] Reddy M.S.B., Ponnamma D., Choudhary R., Sadasivuni K.K. (2021). A Comparative Review of Natural and Synthetic Biopolymer Composite Scaffolds. Polymers.

[33] Sionkowska A. (2011). Current research on the blends of natural and synthetic polymers as new biomaterials: Review. Prog. Polym. Sci..

[34] Suamte L., Tirkey A., Babu P.J. (2023). Design of 3D smart scaffolds using natural, synthetic and hybrid derived polymers for skin regenerative applications. Smart Mater. Med..

[35] Bansal P., Katiyar D., Prakash S., Rao N.G.R., Saxena V., Kumar V., Kumar A. (2022). Applications of some biopolymeric materials as medical implants: An overview. Mater. Today Proc..

[36] Jie I.W.K., Lee K.W.A., Yoon S.E., Song J.K., Chan L.K.W., Lee C.H., Jeong E., Kim J.H., Yi K.H. (2025). Advancements in Clinical Utilization of Recombinant Human Collagen: An Extensive Review. Life.

[37] Ning C., Li P., Gao C., Fu L., Liao Z., Tian G., Yin H., Li M., Sui X., Yuan Z. (2023). Recent advances in tendon tissue engineering strategy. Front. Bioeng. Biotechnol..

[38] Chan B.P., Leong K.W. (2008). Scaffolding in tissue engineering: General approaches and tissue-specific considerations. Eur. Spine J..

[39] Delgado L.M., Bayon Y., Pandit A., Zeugolis D.I. (2015). To cross-link or not to cross-link? Cross-linking associated foreign body response of collagen-based devices. Tissue Eng. Part B Rev..

[40] Mohd Roslan M.R., Mohd Kamal N.L., Abdul Khalid M.F., Mohd Nasir N.F., Cheng E.M., Beh C.Y., Tan J.S., Mohamed M.S. (2021). The State of Starch/Hydroxyapatite Composite Scaffold in Bone Tissue Engineering with Consideration for Dielectric Measurement as an Alternative Characterization Technique. Materials.

[41] Koski C., Onuike B., Bandyopadhyay A., Bose S. (2018). Starch-Hydroxyapatite Composite Bone Scaffold Fabrication Utilizing a Slurry Extrusion-Based Solid Freeform Fabricator. Addit. Manuf..

[42] Duan Q., Liu H., Zheng L., Cai D., Huang G., Liu Y., Guo R. (2023). Novel resorbable bone wax containing β-TCP and starch microspheres for accelerating bone hemostasis and promoting regeneration. Front. Bioeng. Biotechnol..

[43] Ali Akbari Ghavimi S., Ebrahimzadeh M.H., Shokrgozar M.A., Solati-Hashjin M., Abu Osman N.A. (2015). Effect of starch content on the biodegradation of polycaprolactone/starch composite for fabricating in situ pore-forming scaffolds. Polym. Test..

[44] Lee C.-S., Hwang H.S. (2023). Starch-Based Hydrogels as a Drug Delivery System in Biomedical Applications. Gels.

[45] Waghmare V.S., Wadke P.R., Dyawanapelly S., Deshpande A., Jain R., Dandekar P. (2018). Starch based nanofibrous scaffolds for wound healing applications. Bioact. Mater..

[46] Mei S., Roopashree R., Altalbawy F.M.A., Hamid J.A., Ahmed H.H., Naser B.K., Rizaev J., AbdulHussein A.H., Saud A., Hammoodi H.A. (2024). Synthesis, characterization, and applications of starch-based nano drug delivery systems for breast cancer therapy: A review. Int. J. Biol. Macromol..

[47] Shapi’i R.A., Othman S.H., Nordin N., Kadir Basha R., Nazli Naim M. (2020). Antimicrobial properties of starch films incorporated with chitosan nanoparticles: In vitro and in vivo evaluation. Carbohydr. Polym..

[48] Sarhadi H., Shahdadi F., Salehi Sardoei A., Hatami M., Ghorbanpour M. (2024). Investigation of physio-mechanical, antioxidant and antimicrobial properties of starch–zinc oxide nanoparticles active films reinforced with Ferula gummosa Boiss essential oil. Sci. Rep..

[49] Skopinska-Wisniewska J., Wegrzynowska-Drzymalska K., Bajek A., Maj M., Sionkowska A. (2016). Is dialdehyde starch a valuable cross-linking agent for collagen/elastin based materials?. J. Mater. Sci. Mater. Med..

[50] Lee K.I., Olmer M., Baek J., D’Lima D.D., Lotz M.K. (2018). Platelet-derived growth factor-coated decellularized meniscus scaffold for integrative healing of meniscus tears. Acta Biomater..

[51] Baek J., Lee K.I., Ra H.J., Lotz M.K., D’Lima D.D. (2022). Collagen fibrous scaffolds for sustained delivery of growth factors for meniscal tissue engineering. Nanomedicine.

[52] Lee K.I., Gamini R., Olmer M., Ikuta Y., Hasei J., Baek J., Alvarez-Garcia O., Grogan S.P., D’Lima D.D., Asahara H. (2020). Mohawk is a transcription factor that promotes meniscus cell phenotype and tissue repair and reduces osteoarthritis severity. Sci. Transl. Med..

[53] Riaz T., Zeeshan R., Zarif F., Ilyas K., Muhammad N., Safi S.Z., Rahim A., Rizvi S.A.A., Rehman I.U. (2018). FTIR analysis of natural and synthetic collagen. Appl. Spectrosc. Rev..

[54] Salthouse D., Goulding P.D., Reay S.L., Jackson E.L., Xu C., Ahmed R., Mearns-Spragg A., Novakovic K., Hilkens C.M.U., Ferreira A.M. (2024). Amine-reactive crosslinking enhances type 0 collagen hydrogel properties for regenerative medicine. Front. Bioeng. Biotechnol..

[55] Brudzyńska P., Kulka-Kamińska K., Piwowarski Ł., Lewandowska K., Sionkowska A. (2024). Dialdehyde Starch as a Cross-Linking Agent Modifying Fish Collagen Film Properties. Materials.

[56] Tian R., Zhao Y., Fu Y., Yang S., Jiang L., Sui X. (2024). Sacrificial hydrogen bonds enhance the performance of covalently crosslinked composite films derived from soy protein isolate and dialdehyde starch. Food Chem..

[57] Cui T., Sun Y., Wu Y., Wang J., Ding Y., Cheng J., Guo M. (2022). Mechanical, microstructural, and rheological characterization of gelatin-dialdehyde starch hydrogels constructed by dual dynamic crosslinking. LWT.

[58] Yamaoka H., Yamaoka K., Watanabe S., Tanaka H., Hosoyamada M., Komuro Y. (2022). Lactose Stabilization Prolongs In Vivo Retention of Cross-linked Fish Collagen Subcutaneous Grafts in Nude Mice. Plast. Reconstr. Surg. Glob. Open.

[59] Mori H., Shimizu K., Hara M. (2013). Dynamic viscoelastic properties of collagen gels with high mechanical strength. Mater. Sci. Eng. C Mater. Biol. Appl..

[60] Omobono M.A., Zhao X., Furlong M.A., Kwon C.-H., Gill T.J., Randolph M.A., Redmond R.W. (2015). Enhancing the stiffness of collagen hydrogels for delivery of encapsulated chondrocytes to articular lesions for cartilage regeneration. J. Biomed. Mater. Res. Part A.

[61] Lin H., Zhao Y., Sun W., Chen B., Zhang J., Zhao W., Xiao Z., Dai J. (2008). The effect of crosslinking heparin to demineralized bone matrix on mechanical strength and specific binding to human bone morphogenetic protein-2. Biomaterials.

[62] Nazarzadeh Zare E., Khorsandi D., Zarepour A., Yilmaz H., Agarwal T., Hooshmand S., Mohammadinejad R., Ozdemir F., Sahin O., Adiguzel S. (2024). Biomedical applications of engineered heparin-based materials. Bioact. Mater..

[63] Ikegami Y., Mizumachi H., Yoshida K., Ijima H. (2020). Heparin-conjugated collagen as a potent growth factor-localizing and stabilizing scaffold for regenerative medicine. Regen. Ther..

[64] Wu M., Sapin-Minet A., Stefan L., Perrin J., Raeth-Fries I., Gaucher C. (2025). Heparinized collagen-based hydrogels for tissue engineering: Physical, mechanical and biological properties. Int. J. Pharm..

[65] Wang W., Rigueur D., Lyons K.M. (2014). TGFβ signaling in cartilage development and maintenance. Birth Defects Res. Part C Embryo Today Rev..

[66] Du X., Cai L., Xie J., Zhou X. (2023). The role of TGF-beta3 in cartilage development and osteoarthritis. Bone Res..

[67] Chen Y., Mehmood K., Chang Y.-F., Tang Z., Li Y., Zhang H. (2023). The molecular mechanisms of glycosaminoglycan biosynthesis regulating chondrogenesis and endochondral ossification. Life Sci..

[68] Zhao G.Z., Zhang L.Q., Liu Y., Fang J., Li H.Z., Gao K.H., Chen Y.Z. (2016). Effects of platelet-derived growth factor on chondrocyte proliferation, migration and apoptosis via regulation of GIT1 expression. Mol. Med. Rep..

[69] Tamaddon M., Wang L., Liu Z., Liu C. (2018). Osteochondral tissue repair in osteoarthritic joints: Clinical challenges and opportunities in tissue engineering. Bio-Des. Manuf..

[70] Chiang K.-Y., Matsumura F., Yu C.-C., Qi D., Nagata Y., Bonn M., Meister K. (2023). True Origin of Amide I Shifts Observed in Protein Spectra Obtained with Sum Frequency Generation Spectroscopy. J. Phys. Chem. Lett..

