# Supplementary material for: Dialdehyde Starch Cross-Linked Collagen with Heparin Conjugation: Characterization and Feasibility Study for Osteochondral Tissue Repair
Source: Gels. 2025 Oct 24;11(11):850. doi: 10.3390/gels11110850 (PMC12652762; doi:10.3390/gels11110850)
Supplement: Supplementary file 1 [file gels-11-00850-s001.zip › Supplemental materials_Rev_Final.pdf]

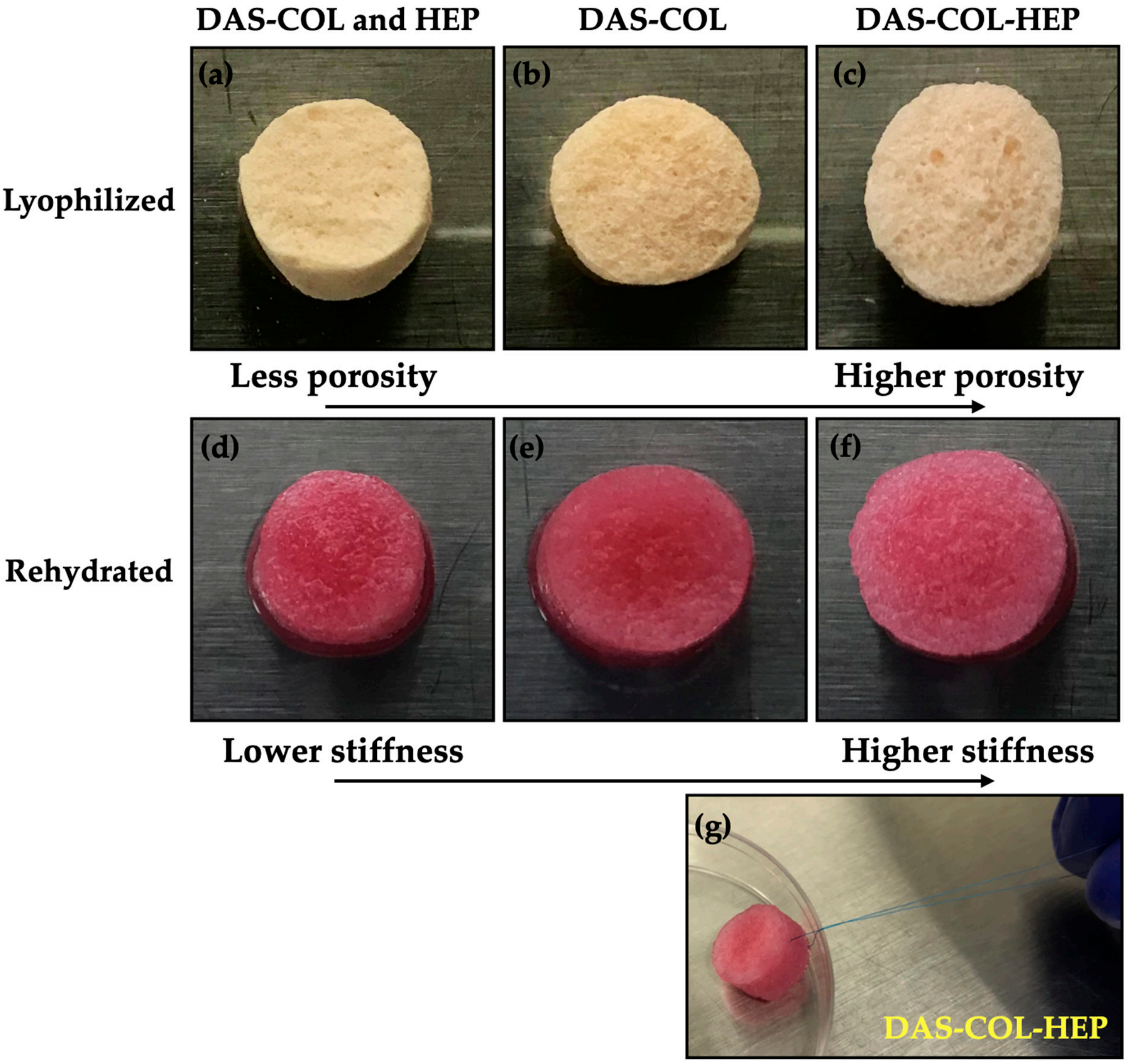

Figure S1. DAS-COL-HEP with different porosity and stiffness under various heparin conjugation conditions: (a) Heparin mixed with post-crosslinked DAS-COL (DAS-COL and HEP); (b) DAS-COL without heparin conjugation (DAS-COL); (c) Heparin mixed during DAS-COL preparation, followed by crosslinking during reconstitution and neutralization steps (DAS-COL-HEP); (d) Rehydrated DAS-COL and HEP; (e) Rehydrated DAS-COL; (f) Rehydrated DAS-COL-HEP; (g) Suturable DAS-COL-HEP after rehydration: Collagen gel (4% w/v collagen), 5% w/v DAS, 0.1% w/v heparin, reconstitution buffer, and 0.75N NaOH neutralization buffer were used for the testing. DMEM media was used for rehydration. The presence of phenol red in the DMEM media indicates neutralized or weak alkaline pH. Only the DAS-COL-HEP sample could be sutured effectively. Porosity and stiffness were higher in DAS-COL-HEP samples compared to other formulations, suggesting that heparin should be mixed during DAS-COL preparation at an acidic pH, followed by neutralization to initiate the crosslinking reaction.

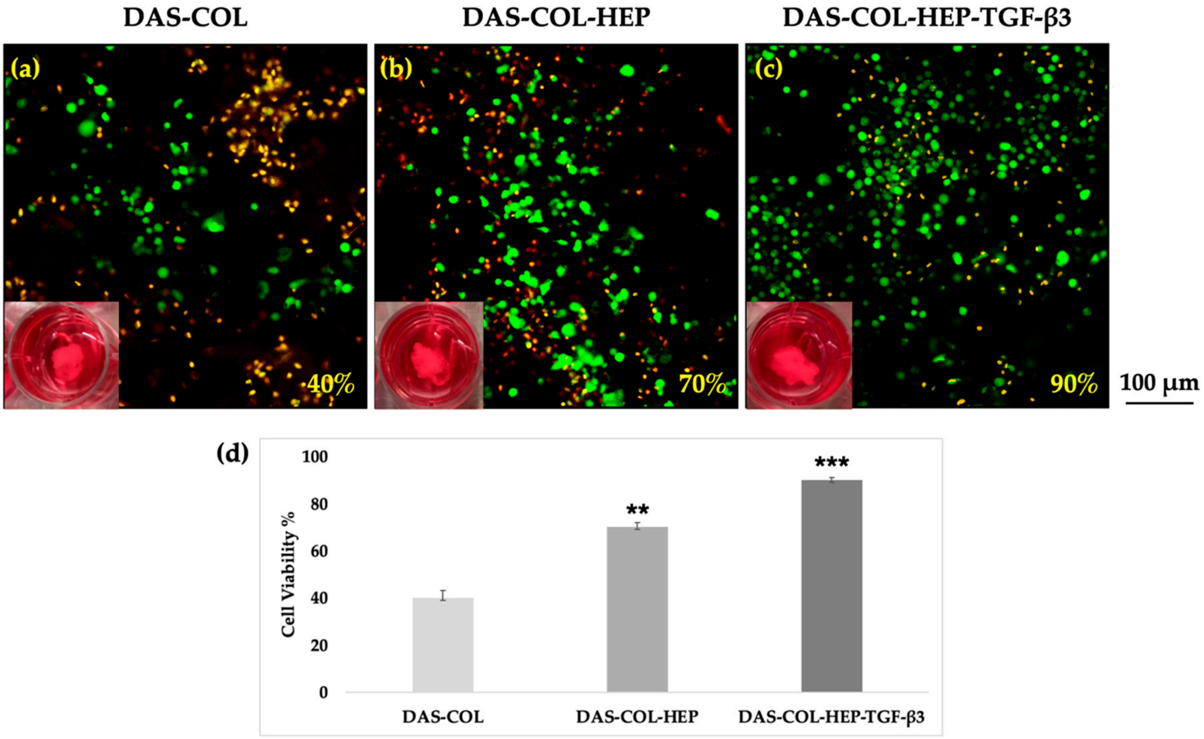

Figure S2. Pilot experiment of co-cultured bovine chondrocytes ($1x10^7$ cells/ml, n=3) with different DAS-COL gel environments over 3 days: (a) Chondrocytes in DAS-COL gel (40% cell viability); (b) chondrocytes in DAS-COL-HEP gel (70% cell viability); (c) chondrocytes in DAS-COL-HEP-TGF-β3 (90% cell viability); (d) quantitative cell viability analysis of each comparison group. ** $p < 0.01$, *** $p < 0.001$ (one-way ANOVA) as compared to the DAS-COL group. The cocultured cell-gel mixture (D+3) for each experimental group is shown as a multi-well plate image.

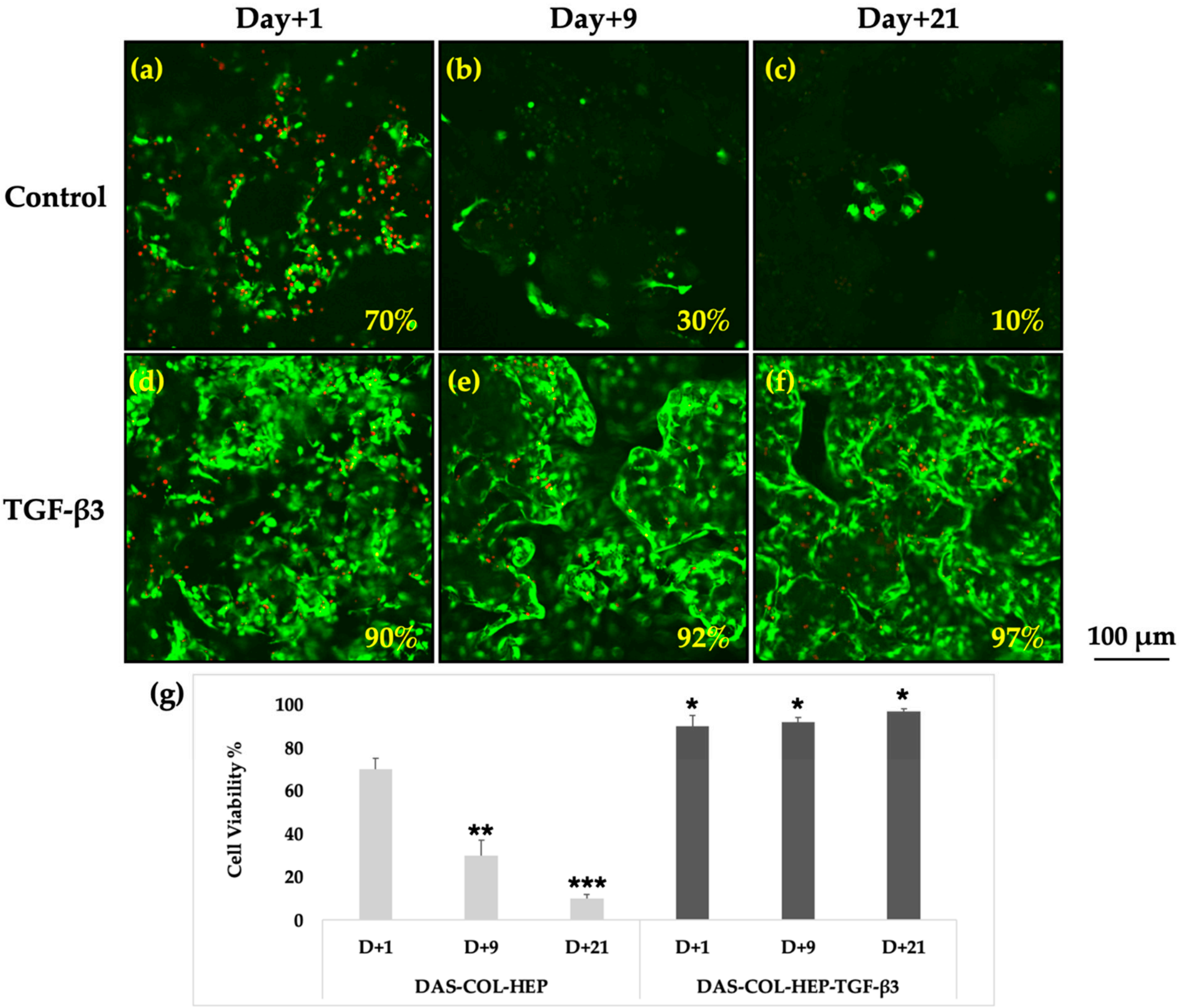

Figure S3. Co-cultured bovine chondrocytes ($1x10^7$ cells/ml, n=3) in DAS-COL-HEP gels with or without TGF-β3 (200ng/mL): (a) Control group (gels without TGF-β3) at Day+1 (70% cell viability); (b) control group at Day+9 (30% cell viability); (c) control group at Day+21 (10% cell viability); (d) Growth factor group (gels with TGF-β3) at Day+1 (90% cell viability); (e) growth factor group at Day+9 (92% cell viability); (f) growth factor group at Day+21 (97% cell viability); (g) quantitative cell viability analysis of each comparison group. * $p < 0.05$, ** $p < 0.01$, *** $p < 0.001$ (one-way ANOVA) as compared to the DAS-COL-HEP at D+1.
